# Hyperthermia versus Oncothermia: Cellular Effects in Complementary Cancer Therapy

**DOI:** 10.1155/2013/672873

**Published:** 2013-04-14

**Authors:** Gabriella Hegyi, Gyula P. Szigeti, András Szász

**Affiliations:** ^1^Department of Complementary and Alternative Medicine, University of Pécs, Hungary; ^2^Department of Physiology, University of Debrecen, and Institute of Human Physiology and Clinical Experimental Research, Semmelweis University, Hungary; ^3^Department of Biotechnics, St. Istvan University, Gödöllő, Hungary

## Abstract

Hyperthermia means overheating of the living object completely or partly. Hyperthermia, the procedure of raising the temperature of a part of or the whole body above normal for a defined period of time, is applied alone or as an adjunctive with various established cancer treatment modalities such as radiotherapy and chemotherapy. However, hyperthermia is not generally accepted as conventional therapy. The problem is its controversial performance. The controversy is originated from the complications of the deep heating and the focusing of the heat effect. The idea of oncothermia solves the selective deep action on nearly cellular resolution. We would like to demonstrate the force and perspectives of oncothermia, as a highly specialized hyperthermia in clinical oncology. Our aim is to prove the ability of oncothermia to be a candidate to become a widely accepted modality of the standard cancer care. We would like to show the proofs and the challenges of the hyperthermia and oncothermia applications to provide the presently available data and summarize the knowledge in the topic. Like many early stage therapies, oncothermia lacks adequate treatment experience and long-range, comprehensive statistics that can help us optimize its use for all indications.

## 1. Introduction

In oncology, the term “hyperthermia” refers to the treatment of malignant diseases by administering heat in various ways. Hyperthermia is usually applied as an adjunct to an already established treatment modality (radiotherapy and chemotherapy), where tumor temperatures in the range of 40–46°C are aspired. Interstitial hyperthermia, hyperthermic chemoperfusion, and whole-body hyperthermia are still under clinical investigation, and a few positive comparative trials have already been completed. In parallel to clinical research, several aspects of heat action have been examined in numerous preclinical studies.

 The traditional *hyperthermia* is controlled the only single thermodynamic intensive parameter, with the temperature. *Oncothermia*, which is a “spin-off” form of the hyperthermia, is based on the paradigm of the energy-dose control, replacing the single temperature concept [1]. With this approach, oncothermia returned to the gold standards of the dose concepts in medicine; instead of the parameter, which cannot be regarded as dose (the temperature does not depend on the volume or mass), oncothermia uses the energy (kJ/kg [=Gy]) like the radiation oncology uses the same (Gy) to characterize the dosing of the treatment.

This paper deals with discussions concerning the direct and indirect cytotoxic effect of heat and energy; heat-and energy-induced alterations of the tumor microenvironment; synergism of heat in conjunction with radiation and drugs; the presumed cellular effects of hyperthermia and oncothermia including the expression of heat-shock proteins (HSPs), induction and regulation of apoptosis, signal transduction, and modulation of drug resistance by hyperthermia or oncothermia.

## 2. Biophysical and Metabolic Differences between Healthy and Cancerous Cells

 The clue to find the mechanisms, which could create the requested optimization, selection and control of the energy intake based on the clear biophysical differences between healthy and cancerous cells, finding the biophysical property to focus the energy on the desired cellular membranes. The main difference could be found in the metabolic processes of malignant cells and their healthy counterparts. In consequence of the physical differences, the malignant cell are distinguishable by their biophysical parameters. The electric properties of the cancerous cells differ from normal, and the main differences are.

- The efficacy of the ATP production in cancerous cell is low. The large ATP demand for the proliferative energy consumption allows less ATP for active membrane stabilization by K^+^ and Na^+^ transport; so the membrane potentiating weakens.
- The cellular membrane of cancerous cells is anyway electrochemically also different from the normal, as well as its charge distribution also deviates.
- The membrane of the cancerous cell differs in its lipid and sterol content form their healthy counterpart.
- The membrane permeability is changed by the previous differences. In consequences of these, the efflux of the K^+^, Mg^2+^, and Ca^2+^ ions, while the efflux of Na^+^ decreases together with the water transport from the cell. Consequently the cell swallows, its membrane potential decreases further. (The efflux of K^+^ regulates the pH of the cell and takes the protons out form the cytosol.) The concentration of Na^+^ increases in the cytosol, and parallel of this, the negative ion-concentration also grows on the glycocalix shell, decreasing the membrane potential and the tumor will be negatively polarized in average. This fact was well used for direct current treatment (electrochemical cancer therapy (ECT)) by Nordenstrom and others.
- The conductivity and the dielectric constant of the tumor tissue will be higher than normal.

The healthy cells work collectively, and their energy-consumption as well as their life-cycles and the availability of resources are controlled on collective way by the various form of the self-organizing. The healthy cells are organized in this way, and their standard cycles, reactions, and structures are complexly regulated in both internal and external areas. 

The malignant cells behave in completely different way. This process is the “ancient” chemical reaction, which was characteristic at the beginning of the evolution of the life, when the oxygen, the general electron acceptor, was available only in a small amount in the atmosphere. It is the fermentative way to utilize the energy of glucose converts it into lactic acid (*CH *_3_ *CHOHCOOH*), producing 2 ATPs in one cycle, while the healthy cycle (Krebs cycle) produces 36 ATPs [2].

The enormously large demand of malignant cells for nutrients, not only the ionic density in the surrounding, makes the malignant cells distinguishable, but their structure is also very different. Their demand for the huge energy production pushes them for the permanent competition for the sufficient amount of nutrition. The cells became independent competitors instead of the normal cooperation, and the harmony had been replaced by autonomy. The intercellular contacts in healthy network are broken in cancer, the order of the tissue had been lost, and the “social signal” between the cells vanished. The malignant cells become disordered, nonconnected, and isolated. The malignancy has definitely different structure that its healthy counterpart, and its dielectric constant and optical refractive index deviates from normal. The lost intercellular contacts block the natural control over the structural changes, and the disordered structure changes the optical properties also [2].

## 3. Hyperthermia

Hyperthermia is the process of raising the body temperature, either locally or globally, for medicinal purposes. Historically, hyperthermia has been recognized for its curative power in treating tumors [3, 4]. The first application of hyperthermia for regional cancer control dates to 1898 when the Swedish gynecologist Westermark [5] treated cervical cancer by running hot water through an intracavitary spiral tube. He noted excellent clinical response in the seven patients treated. More recent clinical studies have shown that the success of a hyperthermia cancer treatment is related to the minimum temperature rise occurring in the tumour tissue [6, 7]. Unfortunately, prolonged exposure of normal, healthy tissue to elevated temperatures has a detrimental effect [8].

### 3.1. The Basic Concept

To reach temperatures clearly above the systemic temperature of 37.5°C in a defined target volume is a technical challenge. The effectiveness of hyperthermia treatment is related to the temperature achieved during the treatment, as well as the length of treatment and cell and tissue characteristics. To ensure that the desired temperature is reached, but not exceeded, the temperature of the tumour and surrounding tissues is monitored throughout the hyperthermia procedure. Another important challenge was to minimize damage to healthy tissue and other adverse effects, and physicians carefully monitor the temperature of the affected area. The goal is to keep local temperatures under 44°C to avoid damage to surrounding tissues, and the whole body temperatures under 42°C, which is the upper limit compatible with life. 

Most clinical hyperthermia systems operate by causing a target volume of tissue to be exposed to electromagnetic or ultrasound radiation. A structure is needed that is capable of transferring energy into biological tissue and getting the best approximation of the area to be treated by 3D distribution of SARs. The majority of the hyperthermia treatments are applied using external devices (applicators), employing energy transfer to the tissue [9, 10]. For a heating system to be effective, it must be able to produce final time and temperature histories that include a set of tumor temperatures that can be maintained for long enough times to result in clinically effective thermal doses without also producing unacceptable normal tissue temperatures [11].

A great deal of current research focuses on precisely positioning heat-delivery devices (catheters, microwave and ultrasound applicators, etc.) using ultrasound or magnetic resonance imaging, as well as developing new types of nanoparticles that make them particularly efficient absorbers while offering little or no concerns about toxicity to other tissues. Applicators are positioned around or near the appropriate region, and energy is focused on the tumor to raise its temperature. To describe the different applicators and hyperthermia methods is not our aim in this paper; however, the summary of the effects of regional especially the locoregional hyperthermia on cell and tissue level is in the next chapters. Regional hyperthermia heats a larger part of the body, such as an entire organ. Usually, the goal is to weaken cancer cells so that they are more likely to be killed by radiation and chemotherapeutic medications. 

### 3.2. Mechanisms of Hyperthermia

The cellular effect of hyperthermia is more complicated. Briefly, hyperthermia may kill or weaken tumor cells, and is controlled to limit effects on healthy cells. Tumor cells, with a disorganized and compact vascular structure, have difficulty dissipating heat. Hyperthermia may therefore cause cancerous cells to undergo apoptosis in direct response to applied heat, while healthy tissues can more easily maintain a normal temperature. Even if the cancerous cells do not die outright, they may become more susceptible to ionizing radiation therapy or to certain chemotherapy drugs, which may allow such therapy to be given in smaller doses. Intense heating will cause denaturation and coagulation of cellular proteins, rapidly killing cells within the targeted tissue. A mild heat treatment combined with other stresses (excitation of the appropriate signal pathways) can cause cell death by apoptosis. There are many biochemical consequences to the heat shock response within the cell, including slowed cell division and increased sensitivity to ionizing radiation therapy. Hyperthermia can kill cells directly, increasing blood flow to the warmed area, perhaps doubling perfusion in tumors, while increasing perfusion in normal tissue by ten times or even more [12]. This enhances the delivery of medications. Hyperthermia also increases oxygen delivery to the area, which may make radiation more likely to damage and kill cells, as well as preventing cells from repairing the damage induced during the radiation session. 

While it is acknowledged that at the tissue, organ, and whole body level there are numerous effects that can contribute to the clinical uses of hyperthermia, this review focuses mainly on effects at the cellular level. The goal of hyperthermia research is to find the molecular mechanisms by which heat kills tumour cells or any cells and the mechanisms by which hyperthermia radiosensitizes cells to radiation or chemotherapeutic agents. A major problem is that hyperthermia causes a large number of macromolecular changes and affects functions in all cellular compartments at temperatures above 43°C. 

The potential importance of the hyperthermia for cancer treatment has been highlighted by Coffey et al. [13, 14]. Specifically, the review addresses four topics: (1) hyperthermia induced cell killing, (2) vascular, (3) cellular and intracellular mechanisms of thermal effects in the hyperthermia temperature range, and (4) effects on proteins that contribute to resistance to other stresses, for example, DNA damage.

### 3.3. Vascular Changes of Thermal Effects in the Hyperthermia

Mild heat temperatures cause an increase in blood flow. At the same time, there is a corresponding increase in the oxyhaemoglobin saturation of the individual red blood cells within tumour microvessels. These changes will lead to an overall increase in oxygen availability and improve tumour oxygenation status [15, 16]. At higher temperatures one may see a very transient increase in blood flow during the heating period, but vascular damage soon begins to occur and this will rapidly lead to a decrease in tumour blood flow [17–19]. With higher heat temperatures, there is also a corresponding decrease in oxyhaemoglobin saturation, and these changes will result in a decrease in overall oxygen availability. This lack of oxygen will also give rise to a decrease in tumour pH and ultimately lead to ischemia and cell death [17]. The degree of vascular shutdown at the higher temperatures appears to be dependent on the time and temperature of heating and the tumour type. Normal tissues typically show a very different vascular response to heat, with flow essentially increasing as the temperature increases, even at temperatures that produce substantial vascular occlusion in tumours [20, 21].

### 3.4. Thermal Cell Killing and Other Cellular Effects of Hyperthermia

In the hyperthermia, the main effects at the cellular level that are of relevance to cancer therapy are cell killing. It has been long recognized that hyperthermia in the 40–47°C temperature range kills cells in a reproducible time and temperature dependent manner. In the hyperthermic region there are three particularly significant and important cellular responses for thermal therapy: cytotoxicity, radiosensitization, and thermotolerance [22, 23]. Survival curves for temperatures in the 43–47°C range typically show a shoulder with an exponential reduction in clonogenic survival as a function of time at a given temperature [14]. The “shoulder” of the survival curve reflects a two-step process of cell killing. This is marked by a linear growth arrest in the beginning of heat exposure (reflecting a reversible, nonlethal heat damage), that is followed by exponential cell death. One fundamental observation is that the capability to induce cell death in vitro at lower temperatures <42–43°C is markedly lower than above 43°C [14].

The changes induced by hyperthermia at the cellular level must be due to temperature-induced alterations in molecular pathways, which usually involve inhibition of DNA, RNA, and protein synthesis [23]. However, while protein synthesis is inhibited during heating at higher temperatures, at milder temperatures and after return to normal growth temperature the induction of heat shock proteins occurs [24], which is an inducing event and closely associated with the induction of thermotolerance. Thus, heat shock can lead to both inactivating and activating responses. A fundamental question is the identity of the molecular events leading to the cellular sensing of altered temperature with subsequent alterations in cellular function. The principal conclusion from these studies is that for hyperthermia, thermal dose is a combination of time and temperature.

The intensity of cell death in hyperthermia is showed cell cycle dependence. In general, high heat sensitivity can be observed during the S and M phases. Microscopic examinations of M-phase cells subjected to hyperthermia show damage of their mitotic apparatus leading to inefficient mitosis and consecutive polyploidy. S-phase cells are also sensitive to hyperthermia, where chromosomal damage is observed. Both S- and M-phase cells undergo a “slow mode of cell death” after hyperthermia, whereas those exposed to heat during G1-phase are relatively heat resistant and do not show any microscopic damage. Cells during G1-phase may follow a “rapid mode of death” immediately after hyperthermia. These variations existing between the different cell cycle phases indicate the possible diversity of molecular mechanisms of cell death following hyperthermia [24–26].

The thermal reactions promote numerous other effects than simple cell-killing. The most important for this complementary therapy are those effects that alter the resistance of cells to radiation and/or chemotherapeutic agents. The challenge is to determine which molecular changes are critical for the relevant endpoint such as tumour cell killing and sensitization to chemo- and radiation therapy [3, 27].

To answer the question, there is an unfolding story of tumour pH and its consequences have become clearer over the last two decades [2]. Using new intracellular methods the intra- and/or extracellular pH is measurable in tissues. Under many conditions it has now been confirmed that the intracellular pH in tumour cells is neutral to alkaline as long as tumours are not oxygen and energy deprived [28]. Tumour cells have mechanisms for exporting protons into the extracellular space. It means that a strong pH gradient exists across the cell membrane in tumours (pH_i_ > pH_e_). Interestingly, this gradient is the reverse of that found in normal tissues where pH_i_ is lower than pH_e_ [9, 29, 30]. Under anaerobic condition cancer cells intensively split glucose to lactic acid, and other relevant pathogenetic mechanisms yielding an intensified tissue acidosis are based on substantial ATP hydrolysis, glutaminolysis, ketogenesis, and CO_2_/carbonic acid production. Acidosis can sensitise tumour tissue to single heat treatments and to fractionated hyperthermia. Summarising the relevant data, it can be stated that tumour temperatures >42.5°C, and appropriate heating can reduce both intracellular and extracellular pH, which may further sensitise tumour cells to hyperthermia in the sense of a positive feedback mechanism [28]. Relevant pathogenetic mechanisms leading to an intensified acidosis upon heat treatment (which is reversible after hyperthermia) are

1. an increased glycolytic rate with accumulation of lactic acid,
2. an intensified ATP-hydrolysis,
3. an increased ketogenesis with accumulation of acetoacetic acid and *β*-hydroxybutyric acid,
4. an increase in CO_2_ partial pressures,
5. changes in chemical equilibria of the intra- and extracellular buffer systems (DpH/DT = −0.016 pH units/C),
6. an inhibition of the Na^+^/H^+^ antiporter in the cell membrane [3, 31].

The ATP decline observed upon heat treatment is mostly due to

1. an increased ATP turnover rate (i.e., intensified ATP hydrolysis), and as a result of an increased ATP degradation, an accumulation of purine catabolites has to be expected together with a formation of H^+^ ions and reactive oxygen species at several stages during degradation to the final product uric acid,
2. a poorer ATP yield as a consequence of a shift from oxidative glucose breakdown to glycolysis [28].

The significance of the baseline pH_e_ and pH_i_ values and changes occurring during hyperthermia in clinical studies still remains unclear, since—in contrast to in vitro and in vivo data derived from experimental tumours treated with hyperthermia alone—the results are equivocal so far. Further clarification of these effects is urgently needed.

Thermal sensitivity has been shown to greatly depend on the efficacy of tumour blood flow and parameters defining the metabolic microenvironment such as hypoxia, acidosis, substrate deprivation, accumulation of metabolic waste products, and energy depletion. If recent experimental data are critically evaluated, there is some evidence that besides microcirculatory function, intracellular pH and the bioenergetic status may be decisive factors ultimately modulating the thermosensitivity of cancer cells.

Israël and Schwartz [2] in 2011 summarized the metabolism of cancer cells. Briefly, in tumour cells the glycolysis is elevated, but a pyruvate kinase (PK) “bottleneck” interrupts phosphoenol pyruvate (PEP) to pyruvate conversion. Thus, alanine following muscle proteolysis transaminates to pyruvate, feeding lactate dehydrogenase, converting pyruvate to lactate (Warburg effect) and NAD^+^ required for glycolysis. Cytosolic malate dehydrogenase also provides NAD^+^ (in OAA to MAL direction). Malate moves through the shuttle giving back OAA in the mitochondria. Below the PK-bottleneck, pyruvate dehydrogenase (PDH) is phosphorylated (second bottleneck). However, citrate condensation increases; acetyl-CoA will thus come from fatty acids b-oxydation and lipolysis, while OAA sources are via PEP carboxy kinase and malate dehydrogenase (pyruvate carboxylase is inactive). Citrate quits the mitochondria, (note interrupted Krebs cycle). In the cytosol, ATP citrate lyase cleaves citrate into acetyl CoA and OAA. Acetyl CoA will make fatty acids triglycerides. Above all, OAA pushes transaminases in a direction usually associated to gluconeogenesis! This consumes protein stores, providing alanine (ALA); like glutamine, it is essential for tumors. The transaminases output is aspartate (ASP) it joins with ASP from the shuttle and feeds ASP transcarbamylase, starting pyrimidine synthesis. ASP in not processed by argininosuccinate synthetase, which is blocked, interrupting the urea cycle. Arginine gives ornithine via arginase, and ornithine is decarboxylated into putrescine by ornithine decarboxylase. Putrescine and SAM form polyamines (spermine spermidine) via SAM decarboxylase. The other product 5-methylthioadenosine provides adenine. Arginine deprivation should affect tumors. The SAM destruction impairs methylations, particularly of PP2A, removing the “signaling kinase brake,” PP2A also fails to dephosphorylate PK and PDH, forming the “bottlenecks.” (Black arrows = interrupted pathways.).

Israël and Schwartz [2] suggested some new cancer treatments based on the previously described metabolic advantage of tumour cells.

1. Inhibit alanine transaminase, glutaminase, ornithine decarboxylase, and arginase and decrease alanine, glutamine, arginine, and supplies.
2. Open PK and PDH bottlenecks (enzyme kinase inhibition and PP2A phosphatase activation by methylation helpers).
3. Close citrate synthase, ATP citrate lyase, and increase the NADH mitochondrial potential.
4. Decrease GH/IGF and “tyrosine kinase receptor” signals.
5. Cancel epigenetic changes using HDAC inhibitors and then HAT inhibition
6. Boost immune NK protection.

So important is the steps 1–4 showing strong ATP dependence. The hyperthermia treatment as we described before reduces the intracellular ATP concentration. The major consequence of the low ATP is the inhibition the ATP dependent enzyme activity. 

### 3.5. Effects on Heat Shock Proteins and the Consequent Immunomodulation

Protein damage is the main molecular event underlying the biological effects of hyperthermia in the clinically relevant temperature range (39–45°C). The activation energies for protein denaturation and heat-induced cell death are within the same range [32]. Despite the fact that it is known that protein damage plays a central role in the biological effects of hyperthermia, little is known about what finally kills the cells [33]. Temperature elevations transiently upregulate heat shock genes that encode a class of heat shock proteins (HSPs). The mechanism responsible for the heat shock response is an autoregulatory loop; HSPs normally keep the responsible transcription factor (HSF-1) inactive but upon heating HSP bind with higher affinity to unfolded proteins, triggering the release of HSF-1 from HSP which initiates HSP gene transcription. Once the protein damage/aggregation is restored after the heat shock by the HSP, substrate-free HSP themselves seem involved in attenuating the response by rebinding HSF-1 [34]. As a result, HSP levels transiently rise after heating but also gradually decline again upon prolonged stress free periods. The upregulation of HSP is closely associated with a transient resistant state of cells towards a subsequent second heat shock. It is thought that the elevated HSP levels, by their chaperone activity, protect cells against protein damage induced by the 2nd heating. 

HSPs were originally described with respect to their roles as chaperones induced by temperature shock as well as various other types of stress [12]. HSP are usually divided into small HSP (molecular mass less than 40 kDa) and the HSP 60, HSP 70, HSP 90, and HSP 100 proteins families. Today, at least HSP 27 and HSP 70 are supposed to represent “general survival proteins” that are able to defend cells against a variety of potentially lethal (proapoptotic) stimuli [12]. In hyperthermia, HSPs are thought to be involved in the protection of cells against heat damage. Intracellular HSP synthesis increases when cultured cells are exposed to moderate heat application. At higher temperatures, inhibition of HSP synthesis occurs above a distinct threshold temperature. In general, the temperature, respectively, thermal dose, at which HSP synthesis is inhibited in a given experimental system varies between different cell types, but the respective threshold can be lowered when further (proapoptotic) stimuli are added. As lack of HSP synthesis is associated with exponential cell death, and it is generally accepted that HSPs prevent cells from lethal thermal damage [12, 35–37]. 

Recently, an additional role has been ascribed to HSPs which should be importance in hyperthermia as activators of the immune system. The importance of the HSP family, including HSP70 and HSP90 in immune reactions has been demonstrated, and several researchers have reported that heat-treated tumor cells can play a vaccine-like role and elicit antitumor immunity [12, 35–37]. HSP have been found to play important roles in eliciting potent anticancer immune responses mediated by T-cells, antigen-presenting cells (APCs), and natural-killer (NK) cells. In order to explain the paradoxical situation of how highly conserved HSP mediate cancer immunity, Srivastava et al. [38] proposed the following four paradigms:

- due to short nonconserved immunogenic regions,
- because a tissue specific expression pattern HSP can act as classical foreign antigens,
- HSP could mimic classical T-cell epitopes,
- act as carrier molecules for immunogenic peptides.

Presently, experimental evidence exists for possibilities (a) and (c). Tumour-derived HSP (HSP 70, Hsc70) and gp96 have been shown to chaperone immunogenic peptides to MHC molecules that elicit T-cell responses against primary tumours and metastases. Antigen-presenting cells have been found to be key for mediating this specific immune response. The membrane-bound HSP 70 provides a target recognition structure for transiently plastic adherent NK cells. A correlation of the cytolytic activity of NK cells with the amount of plasma membrane-bound HSP 70 has been determined. Although an HSP 70-NK cell interaction could be demonstrated in binding studies (unpublished data), the mechanism of how NK cells lyse Hsp70 positive tumour target cells remains to be elucidated. HSP 70 expressing tumour cells are more sensitive to lysis mediated by IL-2 stimulated, transiently plastic adherent NK cells, as compared to HSP 70 membrane negative tumour cell. Selectively transiently plastic adherent NK cells, but not T-cells, exhibit an increased cytolytic activity against Hsp70 membrane positive tumour target cells [12, 35–37].

In summary, the data provide evidence that membrane-bound HSP 70 or the extracellular exposed HSP 70 peptide TKD might provide a tumour-selective target recognition structure for CD94 positive NK cells. The elucidation of potential coreceptors or partner molecules of CD94 that mediate triggering signals following contact with HSP 70 protein or HSP 70 peptides is currently under investigation.

## 4. Problem with the Hyperthermia

The requested job is to kill the malignant cells, for what a definite energy dose is necessary [39]. The historical energy-dose-like control (temperature multiplied by its application time) is physically incorrect and operates with an overall energy average in the area, instead of a directed and well-measurable energy dose (measured in kJ). The high energy application could cause controversies the high temperature burns the malignant cells but because it is missing selectivity and it damages the healthy cells and starts unwanted physiological reactions as well as enlarged dissemination possibility. These conditions make the hyperthermia effect not controlled, irrespective that the temperature could be kept on a certain level in the tumor or not. In reality the selectivity which would be necessary for the actions is missing, and the heating attacks all the cells in the target and also unselectively provokes the psychological control and regulation. The original idea of the hyperthermia was the “fire by fire” concept; set a controlled contrafire depleting the possible firesupply, blocking the coming large bush-fire endangering a house. The heated tumor is forced to higher metabolism; high metabolic rate of the cancer lesion is gained by elevated temperature. However, when the surrounding is intact, it delivers the same amount of nutrients than before and does not deliver more glucose for the forced metabolism. The tumor very quickly deflates from nutrients, empties all its energies, suffers, and burns away. However, by the large heating energy heats up the healthy surrounding us well, the blood flow will be enhanced, the nutrients supply will be higher, we make opposite than our aim was to do. The situation became even worst by continuing the high-energy heating: the high blood flow helps the dissemination and could gain the metastases. With this we can definitely worsen the survival and the quality of life of the patient. The blood flow is an important thermodynamic modification factor, which makes the dominant addition to the temperature development in the tumor. According to the fits of measurements, actually the blood flow for tumor and the healthy tissues are both definitely temperature dependent, and in general there is a threshold when the blood flow of the tumor became lower than the blood flow of the healthy surroundings. Below this threshold the efficacy of radiotherapy (which is propositional with the oxygenation of the tissue) and the chemotherapy, which is proportional with the drug-concentration in the tissue) is growing by the increasing of the temperature, because both of these properties are delivered by the increasing blood-flow. However, the complementary therapies over the threshold are suppressed by the decreasing of the blood-flow in the tumor. 

## 5. Change of Paradigm: The Oncothermia

Oncothermia technology heats nonequally; concentrating the absorbed energy to the intercellular electrolytes [40]. This method creates inhomogeneous heating, microscopic temperature differences far from thermal equilibrium. The definitely large temperature gradient between the intra- and extracellular liquids changes the membrane processes and ignites signal pathways for natural programmed cell-death, avoiding the toxic effects of the simple necrosis. The synergy of electric field with the thermal effects potently and selectively makes the job [41].

### 5.1. How the Oncothermia Works, Generally

Advantage of oncothermia, that while the classical artificially focused hyperthermia has to heat up in case of the multiple lesions overlapping all the volume, which contains these lesions; contrary, oncothermia automatically focuses on the lesions in their multiple places, without treating the healthy tissue between. Oncothermia has a simple technical setup. The modulated radiofrequency current (RF) flows through the lesion. The RF current which flows through the cancerous lesion automatically focused by its lower impedance will flow mainly in the extracellular electrolyte, because the cells are electronically isolated by their membrane by more than one-million V/m field strength. The membrane disruption is one of the targeted aims, and many research groups are dealing with the electric field action on the cellular divisions. The main advantage of the electric field application is the missing control of the organism, physiology control over this effect; no physiology feedback is directly limiting the electric field and only its consequences could be regulated. The process made by oncothermia has its main energy delivery into the extracellular liquid, heating it up, and creating a little (1/1000°C) difference between the inner and outer temperature of the cell. This looks only a small difference, but regarding of the very tiny membrane layer (5 nm), the small difference in standard conditions is pretty high: ~200,000°C/m! This starts a prompt heat flow from outside to the cell through the membrane and permanently acts till the temperature difference exists. Despite of the quick heat-flow through this tiny membrane, the heat current is long-lasting, till the full cellular interior is not heated up to the same temperature than outside [42–44]. 

Oncothermia does not require high temperature for the treatment, the energy used for distortion of the selected malignant cells is that of thermodynamic effects (heat-flows are applied instead of the general average isotherms). Oncothermia is based on the modulated electric field effect, which works in synergy of the classical temperature-based hyperthermia concept. In preclinical conditions (in vivo and in vitro) many measurements were done in animals and there are many interested users who tried up till now the temperature development by the method, which is a complex, invasive measurement approach. A well-controlled clinical temperature measurement by the CT-guided fluoroptic sensor showed the temperature increase facility of oncothermia also [44].

However, the main advantage of oncothermia is that the nonequilibrium heating creates a heat-flow through the membrane into the cytosol, which is active till the equilibrium is reached. This gains the efficacy and the reliability of the treatment. The membrane effect by electric field was expected having a thermal limit. It was questioned, and later it was rigorously shown that the effect of oncothermia is not limited in this issue. To enhance the selection for membrane distortion, and enhance the collective (apoptotic) control on the cell-death, a special modulation is built in the oncothermia process [45]. 

The not so high radiofrequency (13.56 MHz) is absorbed in all the electrolytes, but the main energy absorption is in the membrane and the extracellular electrolyte. This creates an extreme SAR between the cells, which makes temperature gradient through the membrane. The treated tissue will be inhomogenicly heated, and heat flows from extracellular to cytosol through the membrane, accompanying with definite other thermodynamical and chemical changes. These definite thermal currents will be continued till the extra- and intracellular temperature reaches equilibrium; so the intracellular electrolyte had been heated up to the equal level.

### 5.2. Oncothermia Is Microscopic Heating of Nanoscopic Targets

The general idea of microscopic heating is simple; the heating energy is not liberated in a sudden single step, but regulated and multiple small, microscopic energy liberation makes the same job. In our case the forwarded energy targets the most influential areas selectively. Instead of the high, general energy pumping into the lesion, the energy is liberated at the membranes of the malignant cells, in a nanometer range. Anyway, the microscopic effect, instead of the large energy liberation, is one of the most update thinking in energy source developments. In the modern technologies, the relatively low efficacy combusting engines are intended to replace by the fuel-cell energy sources and electric motors, which are based on the membrane regulated microscopic reactions of gases. In our case the microenergy liberation at the nanoscopic cellular membrane hits the most sensitive part of the tumor cells. 

The efficacy of the energy depletion intended to pump into the tumor is limited by the energy loss outside of the malignant target. The main factors of the useless energy absorptions are

- the absorbed energy by the tissues transfers the effect to the deep-seated tumor;
- the heat exchange by the blood flow;
- the heat exchange by the heat conduction from the tumor to the surroundings;
- these heat sinks are modifying the overall performance of the treatment and make uncontrolled the full heating process for the malignancy. The real effect which used for the intended treatment is to be less than the losses, and the efficacy is usually less than 25%, which is very low. The problem of this is not only that the large part of energy is wasted, but also the useless energy part could be dangerous by overheating the healthy tissues as well as increasing the metabolic rate and also making physiology answer on this effect which tries to break the homeostasis. The massively heated tumor volume intensifies the control of physiology and weakens the expected effect.

The adequate corrective actions for these challenges would be the more precise targeting, decreasing the losses in the surrounding and avoid the physiological corrections to suppress the desired effect. Construction the solution some new effects have been used increasing the efficacy.

- Apply the electric field as carrier of the energy, and that field cannot be compensated by homeostatic control.
- Apply a correct microscopic targeting, using the energy absorption cell-by-cell efficiently.
- Apply such mechanisms, which initializes natural effects kill the malignant cells.
- Apply mechanism, which carries information for disseminated cells to be blocked.

According to the calculations a relatively small amount of energy can heat up the average-sized tumors to the appropriate temperature, if it targets the tumor accurately enough. The relatively low bioimpedance of malignant lesions makes possible the automatic focusing; the applied RF-current will choose the “easiest” way to flow, automatically loading the tumor with highest current density [46]. 

The approach using electric impedance for quality assurance is partly comparable with the ion concentration concept of ionizing radiation characterization: the old unit measured the ion pair production, 1 R (roentgen) = 2.58 ∗ 10^−4^ C/kg. Note, interestingly, that an electric field application without an increase in temperature (using less than 5W power) has also been found effective against cancer, by using galvanic (DC) current applications, producing ion-pair effects in extracellular electrolyte. This electric tumor treatment measures and controls the tissue resistance and the quality parameter is the applied charge load (in Coulombs, C), with results reported in several peer-reviewed journals and conferences organized on the subject. Yet few studies discuss the biological mechanisms involved in electromagnetic field induced hyperthermia. However, the effect of electric field remains to be a hot topic in science [40]. 

Oncothermia works with much less forwarded energy, by focusing energy directly on the malignant tissue using its impedance selectivity even by cellular resolution. This effect is based on the low impedance of the tumor, due to its metabolism, which is higher than that of its healthy counterpart's. This special focusing in fact makes the treatment safer and really nontoxic. The high and fermentative glucose→2ATP process [3, 47] produces higher ionic concentration in the more active cellular environments and different physiological conditions and allows even spatial resolution by this effect. The real focusing could be measured by RF-current density image, which spectacularly shows the focusing of the current in the tumor. The focusing effect in this meaning is very similar to the PET picture, which shows the enhanced metabolic activity of the tumor.

Model of oncothermia method producing higher efficacy could be demonstrated with a simple parallelism with the solutions of modern energy sources. The conventional engines in vehicles use the energy of explosion of different chemicals (e.g., petrol, diesel, and kerosene). The explosion by a spark or heating over their activation energy liberates large energy in a short time, and only a small fraction of this could be applied beneficially, while most of the energy is radiated, conducted, or lost in various other ways. One of the largest loss is the heat exchange by the high temperature, which somehow has to be used again (e.g., intercooler and turbo). The most modern solution, however, is the set of microscopic explosions, promoting the chemical reactions individually by a membrane control (i.e., fuel cell solution) and using the energy step-by-step as a sum of the microreactions. This model is anyway learned from the living organisms, when the energy is liberated gradually in a “ladder” of multistep processes, and also moderated by surface reactions. 

In this case, the lost energy is minimal; the efficacy of the energy utilization and its control is maximal. The energy is concentrated in this case directly to the chemical reactions and does not involve the previously listed losses. The energy liberated by the microreactions are used for the desired job in full, while the explosion-like, huge energy-supply in short time cannot be optimally used, because the intensity of the immediate offer for the available energy is too much for prompt use. This causes a large demand of waste and a low efficacy of the desired effect. The problem of the heating however shows a false, specious effect of applications in biology. When the liberated energy is not used as active biochemical or biophysical driving-force than the waste appears as a simple growth of the temperature in the target. This deceptive illusion looks as higher efficacy. This process is of course a better heating, wasting the energy and not used for the actually necessary processes, distributing the excess energy in the neighborhood, and gaining the average energy of all the particles in the target. 

 This is a typical loose of aims by illusions: the temperature makes only conditions that are implements and not the aim. The question “Tool or goal?” became relevant to study the temperature alone. By a simple example of mixing the tool and the goal in our everyday life, the graduation is a tool for our professional life, however, when somebody makes regards the certificate of studies as a goal, its application, the aim of the study looses. Mixing the tool with the action creates false goal in hyperthermia application, increasing the temperature alone. This “auto-suggestion” creates such situation that magnetic resonance imaging (MRI) is applied to control the temperature during the treatment, instead of using this capable imaginary method to see what is happening in the tumor indeed. 

The non-temperature-dependent cell destruction is studied by ultrasound and widely by electric fields, which is a hot topic in science [40, 47]. Anther line of the development of energy absorption in depth for killing the malignant cells was parallel: the electromagnetic treatments to cure cancer. The first famous name was D'Arsonval. His method had fantastic popularity at the turn of the 19th-20th centuries. Numerous devices were developed and applied widely, but the expected breakthrough result was missing. The first conference in this field was organized in 1992 in Beijing. In the beginning the galvanic electric current was used to treat cancer. This method had delivered remarkable results; the biological mechanisms involved in electromagnetic field are intensively investigated and the effect of electric field is studied on various side of its complex behavior. Some special treatments are established to direct electric treatment of the tumor (mainly with DC-current), controlling the tissue resistance, and the quality parameter is the applied charge load. Several good surprising results reported and conferences organized on the subject. Yet few studies discuss the biological mechanisms involved in electromagnetic field induced hyperthermia.

**Figure 1:**
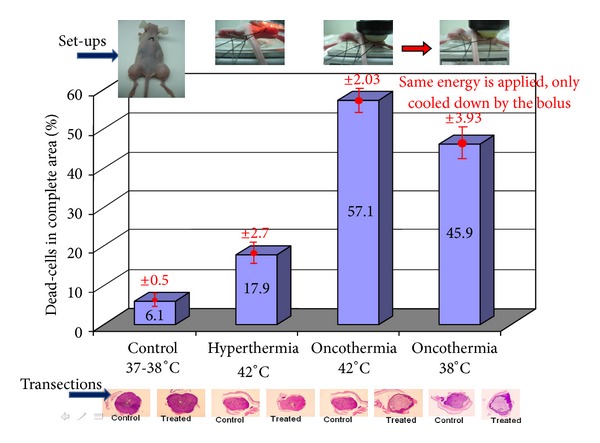
The cell-destruction ability of oncothermia is three times higher than of conventional hyperthermia at the same 42°C temperature (single shot treatment, 30 min). Pumping the same energy as for temperature 42°C, but cooling the lesion by outside water-bolus (to 38°C temperature), the efficacy of the cell-destruction remained much higher in oncothermia than in hyperthermia at 42°C temperature.

 Based on microscopic effects, there not only the heating makes the effect, but the electric field itself has a strong synergy with this (see Figure 1), having significantly larger cell killing in malignancy at 38°C, than the conventional hyperthermia has on 42°C. The process is selective [48]. The RF current is choosing the “easiest” path to flow, and due to the high ionic concentration of the near-neighborhood of malignant cells, the current will be densest at the tumor cells. The experimental results well support this idea. In the case of healthy cells, the load is equal for all the cells, with no difference between the treated and control samples. When we gain the metabolism (immortalized cells) but not yet malignant acceleration, the effect is selectively higher but not significant. However, when the malignancy is present, the cellular growth is aggressive, the selection became effective, and it kills the tumor cells without affecting the healthy ones in the coculture. 

This electric field effect well demonstrates, that the average kinetic energy (temperature) has not decisional effect. The main action is the targeted energy-delivery, which could be done on such low average energy as the standard healthy body temperature. 

**Figure 2:**
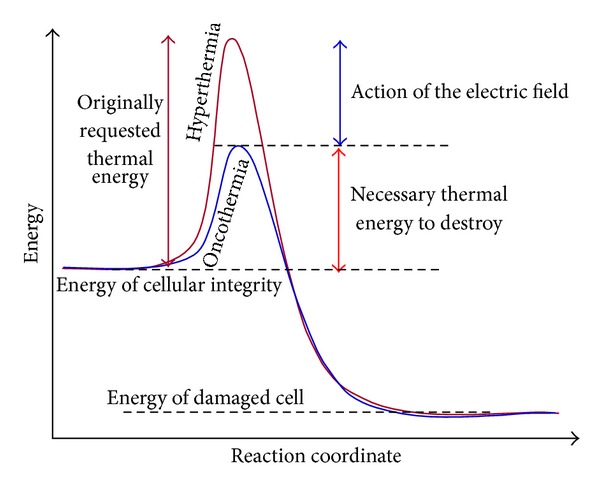
Oncothermia needs less thermal energy to make the same distortion than the classical hyperthermia does. Part of the thermal energy is replaced by the electric field.

The cell killing needs energy, and afterwards the overall energy of the system would be decreased from a well ordered (bounded) state (which was in the case of the living cell) to a disordered chemical state with some broken chemical bonds. The transition from the ordered (chemically higher energy) state to the disordered (chemically lower state) arrangement the well-known gap energy must be pumped. This gap-energy has different components. For hyperthermia the heat energy gives the full energy consumption however, in oncothermia a significant field effect takes part in the distortion mechanism. This simple allows easier cell-destruction by oncothermia (see Figure 2); similar to the well known catalytic reactions. 

The large extracellular SAR makes not only thermal but also electric inhomogenity in the tissue; the extracellular matrix has higher current density than the other electrolytes. The current density gradient is accompanied with the gradient of the electric field, which could reorient the high-dielectric constant proteins in the extracellular liquid. The orientation of these protein molecules would be constrained perpendicular on the membrane surface. By this effect the lost adherent connections could be rebuilt in between the malignant cells, which were indeed shown experimentally. This effect helps to suppress the metastatic dissemination as well as promotes the intercellular signals to activate the natural cell-killing mechanisms. 

### 5.3. Oncothermia Promotes the Programmed Cell-Deaths of Tumor

**Figure 3:**
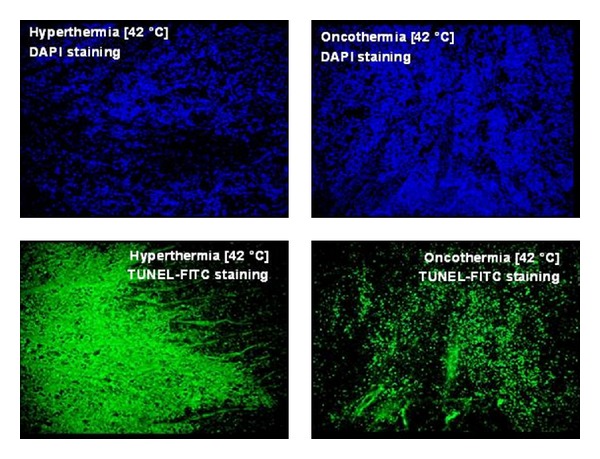
Upper panel: DAPI staining (stains the double strains of DNA only); lower panel: TUNEL-FITC staining (enzymatic label of the strain-break of the DNA). TUNEL is typical for necrosis (diffuse staining) in hyperthermia and for apoptosis (staining of nuclei locations) in oncothermia treatments.

The selection and the tumor destruction of oncothermia became trivial hours later of the treatment. The time delay indicates the long-duration processes, which were identified as programmed cell-death (apoptosis) [49]. Detecting the double strains of DNA (DAPI staining; see Figure 3 upper panel) and measuring the enzymatic labeled strain-breaks of DNA (TUNEL-FICT; see Figure 3 lower panel), the apoptosis is highly likely in oncothermia, while at identical temperature in classical hyperthermia the necrosis is preferred. Consequently, the main effect in oncothermia is the apoptosis contrary to the conventional hyperthermia, which operates mainly by necrosis. Investigating the apoptosis by various methods (morphology, beta-catenin relocation, p53 expression, Connexin 43, Tunel, DNA-laddering, etc.), the effects are indicating the same apoptotic process. This process is non-toxic (no inflammatory reactions afterwards) and promotes the immune reactions (parallel work with these) and not makes processes against those. 

### 5.4. Oncothermia Limits the Dissemination of Malignant Cell

Dissemination is blocked by the sticking of the cells to each other. The adherent connections and the junctions between the malignant cells are reestablished, the cells are connected again, their autonomy is limited, and the possibility of the dissemination decreases. The immune system (with presently not clear mechanism) acts against the metastatic lesions (abscopal, bystander effect), and the local treatment of the primary tumor decreases the distant metastases as well. These effects are the most important for the survival and quality of life. 

The main threat of life by tumorous diseases is the distortion of the organs which are essential for the life of the organism. When the tumor grows somewhere without endanger the important systems like the respiratory system, central nervous system, cardiovascular system, and so forth, it has no life-threatening efect. These tumors are local (benign or early malignant), and effect their elimination is possible. The real life-threatening is the malignancy, when the cells are disseminated from the tumor-lesion by the various transport systems (lymph and blood), or their effect become systemic by one of the general mechanisms of the organism. The problem of the classical hyperthermia is that sometimes it promotes the dissemination of the malignant cells. 

Oncothermia is different from this point of view as well. It blocks the dissemination, avoiding their motility due to the lazy connections to the tumor. Oncothermia makes it by reestablishing the cellular connections, which is also great success to save the life. The built up connections could force not only the sticking together, but makes bridges between the cells for information exchange to limit the individuality, the competitive behavior of the malignant cells. 

**Figure 4:**
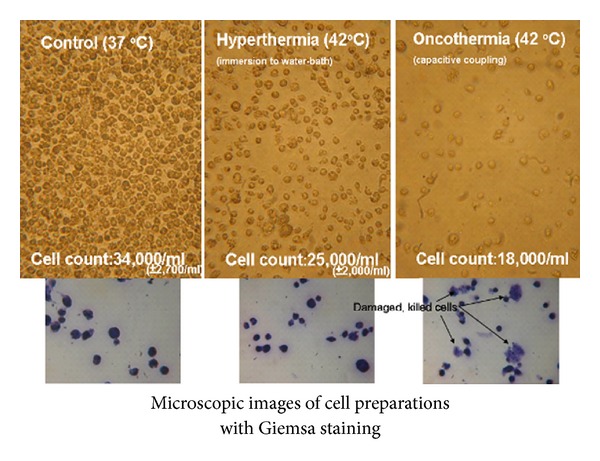
Lethality comparison with traditional hyperthermia in vitro experiments (fixed suspension sample): HL-60 leukemia cell line.

Comparison of hyperthermia and oncothermia combined both methods with mitomycin-c (MMC) single dose chemotherapy in vivo at tissue and cellular level using histological examinations is shown. HT29 human colorectal carcinoma cell line derived xenograft tumor model in nude mouse: 2 animals for hyperthermia (42°C) + 3 mg/kg MMC ip. (30 min before the treatment) and 2 animals for oncothermia (42°C) + 3 mg/kg MMC ip. (30 min before the treatment). The constrained thermodynamic transport effects destabilizes the cell membrane, increases its permeability and could make its bobbling and distortion. These are high efficacy factors favoring oncothermia over its temperature-equivalent hyperthermia counterpart; see Figure 4. It also produces higher concentration of HSPs in the outer membrane and in the extracellular matrix. The higher HSP concentration in the vicinity of the malignant cells together with the changes of the adherent connections between the cells induces apoptosis. 

 The setup made possible a fine temperature control, which allowed to keep the heating, keeping and cooling dynamist also identical. This makes identical heat-shock protein induction by the temperature changes. The temperature-dependent equality was controlled by luciferase transient transfected HEK293 cell lines. Despite the equal temperature curves, oncothermia produces higher concentration of HSPs in the outer membrane and in the extracellular matrix. The higher HSP concentration, in the vicinity of the malignant cells, is one of the factors to induce apoptosis. 

Changes of adherent connections (E-cadherin and *β*-catenin) are also indicators of the gain of the social signals promoting the apoptosis. Remarkable change could be observed on beta-catenin dynamic development by time after the treatment. on HepG2 human hepatocellular carcinoma cell line. This considerable change after 24 hours of the treatment is sharply different from hyperthermia on the same temperature and supports the other observations of the non-temperature-dependent processes. The sudden regrouping the beta-catenin and its enrichment at the cell-nuclei could be an indicator of apoptosis [50, 51].

Many in vitro and in vivo preclinical studies as well as twenty years of entirely positive practice and huge number of retrospective clinical studies are behind of oncothermia [52–57].
